# Unveiling a rare radiological discovery: Interlobar pneumothorax in a young female patient

**DOI:** 10.1016/j.radcr.2023.06.021

**Published:** 2023-06-20

**Authors:** Shorabh Sharma, Shivam Khatri, Akshay Agarwal, Jordan Smith, Swati Sharma, Swati Patel

**Affiliations:** a Division of Internal Medicine, Department of Medicine, St. Barnabas Hospital, Bronx, NY, USA; b CUNY School of Medicine, 160 Convent Ave, New York, NY 10031, USA; c Department of Medicine, NYU Grossman School of Medicine, New York City, NY, USA; d Division of General Internal Medicine, Mount Sinai Morningside, New York City, NY, USA

**Keywords:** Interlobar pneumothorax, Diagnostic radiology, Spontaneous pneumothorax

## Abstract

Pneumothorax is a medical condition characterized by air in the space between the visceral and parietal pleural surfaces, with spontaneous and traumatic classifications. Spontaneous pneumothorax is further divided into primary and secondary groups based on the presence or absence of clinically apparent underlying lung disease. Here we present a case of a 23-year-old female with no past medical history who presented with chest pain and shortness of breath, ultimately diagnosed with primary spontaneous interlobar pneumothorax. Despite advancements in medical diagnosis techniques, spontaneous interlobar pneumothorax remains a rare presentation of pneumothoraces. Treatment strategies for clinically stable patients with a first episode of primary spontaneous pneumothorax include observation with or without supplemental oxygen, with further intervention only if the pneumothorax fails to improve or worsens.

## Introduction

A pneumothorax is a common medical condition defined as air introduced into the space between the visceral and parietal pleural surfaces and can be broadly classified as either spontaneous or traumatic. Spontaneous pneumothorax is traditionally divided into primary and secondary groups by international guidelines; these divisions are based on the absence or presence of clinically apparent underlying lung disease [1,2]. Primary spontaneous pneumothorax is 3-6 times more common in men than in women with an incidence of 7.4 per 100,000 population per year in the United States [1]. Secondary spontaneous pneumothorax is more common in males and in those aged >55 years [1,2]. The prevalence of iatrogenic pneumothorax and interlobar pneumothorax is poorly studied. Spontaneous interlobar pneumothorax is an unusual topographical feature of pneumothoraces.

## Case presentation

A 23-year-old female with no past medical history presented to the emergency room with chest pain and shortness of breath for 2 days. The pain started spontaneously while she was lying in bed. It was described as sharp, central, with radiation to the left chest, and it worsened by deep inspiration. This pain was associated with shortness of breath, wheezing, and mild chills. The patient took a 3-hour flight 1 day prior to the onset of symptoms. She stated that she was involved in a physical altercation the day her symptoms started. On physical exam, no bruising on the chest fault or other injuries to the torso were noted. She denied similar previous episodes or a history of smoking.

**Fig. 1:**
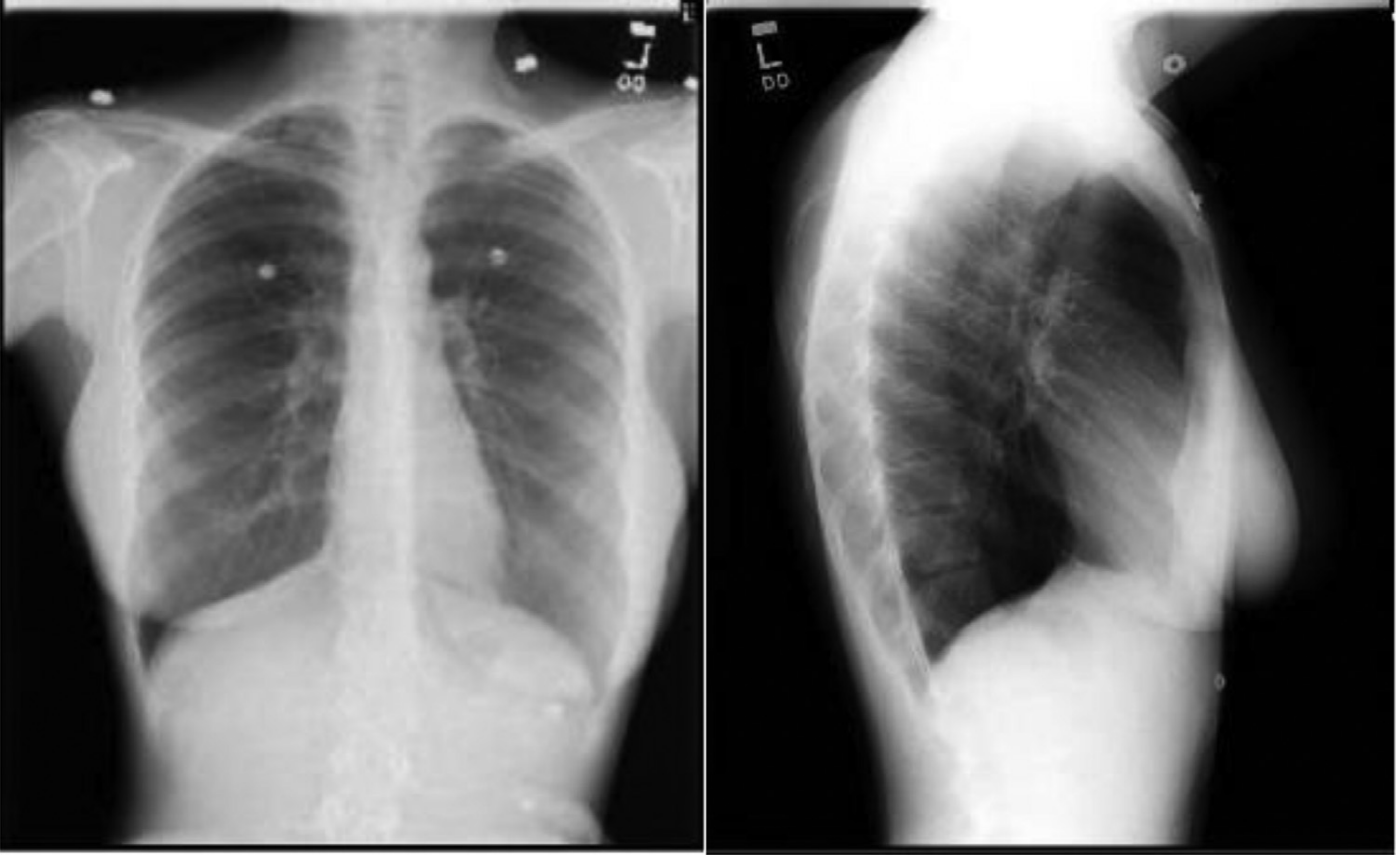
Chest X-ray AP and lateral views - looks within normal limits without any evidence of pneumothorax, effusion or atelectasis.

On initial presentation, her vital signs included a temperature of 99.2°F, a heart rate of 120 beats/min, a blood pressure of 117/78 mm Hg, a respiratory rate of 18 breaths/min and a saturation of 95% on room air. Physical exam revealed an anxious appearing female in no apparent respiratory distress. Cardiopulmonary exam was significant for tachycardia only. There was good air movement with clear and equal lung sounds bilaterally throughout all lung fields. Laboratory results for complete blood count and chemistries were grossly within normal limits. EKG showed sinus tachycardia without right heart strain. Chest X-ray (Fig. 1) was unremarkable, without infiltrates, effusions, or pneumothorax.

**Fig. 2:**
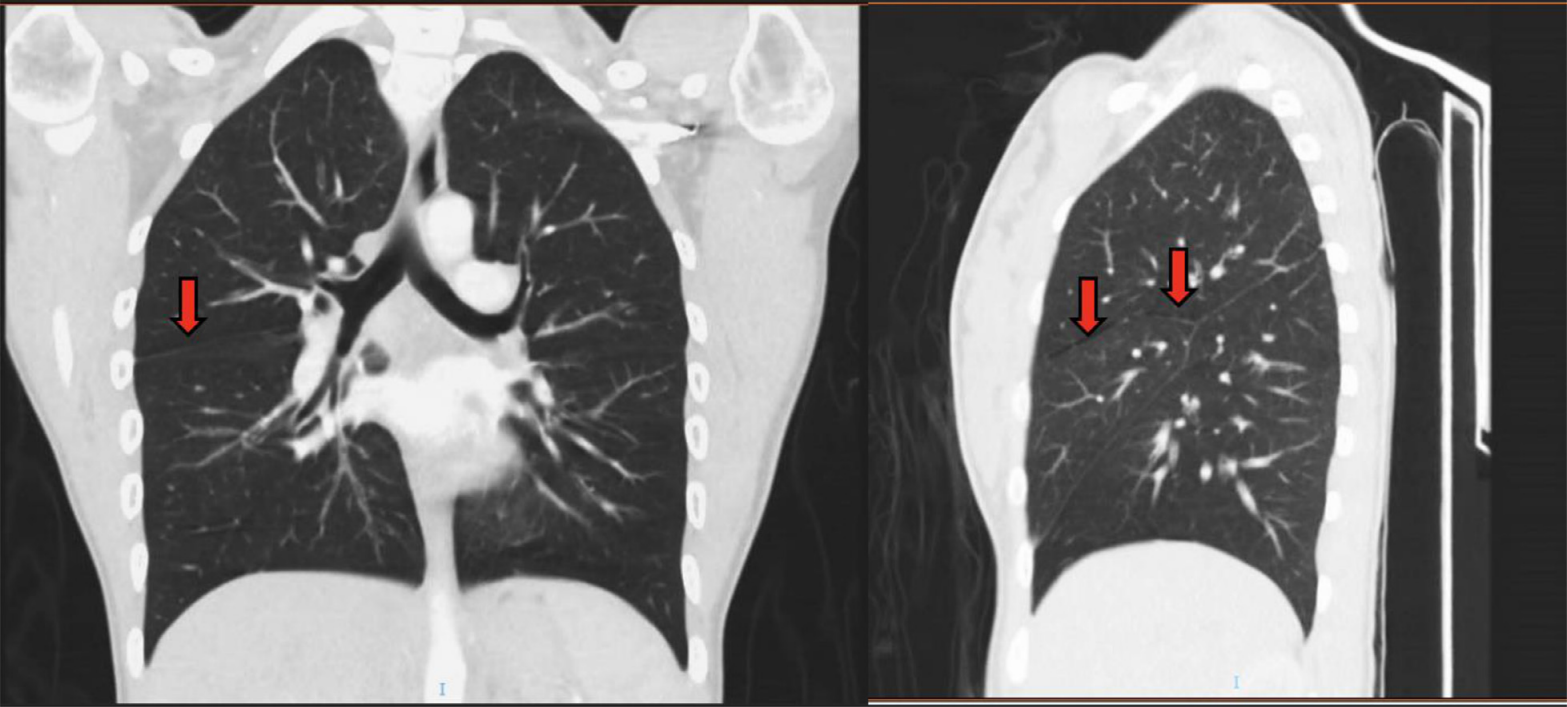
CT angiogram of chest AP and lateral views - curvilinear streak seen immediately adjacent to the course of the minor fissure of the right lung as marked by the red arrow.

**Fig. 3:**
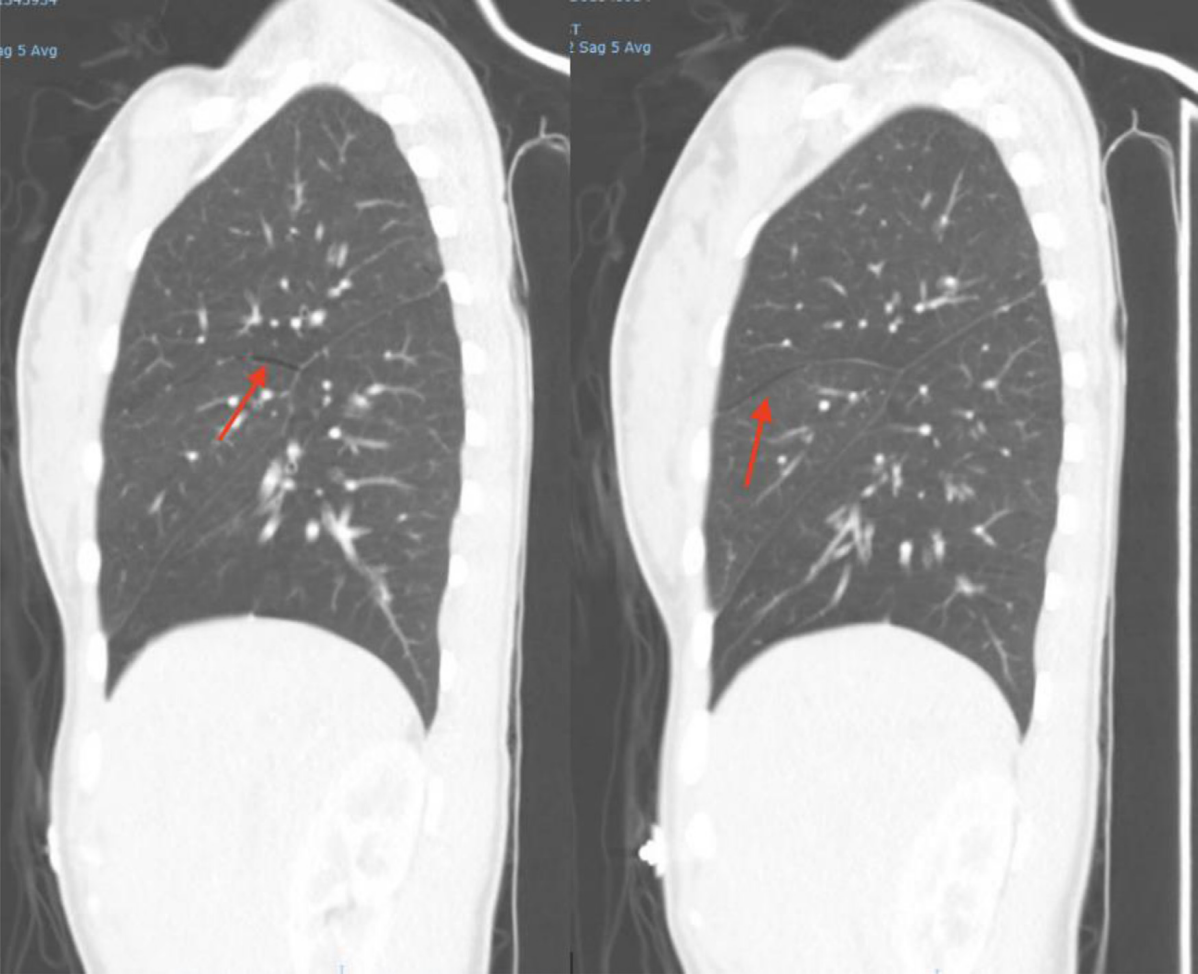
Lateral view of the CT angiogram depicting the hyperlucency of a spontaneous interlobar pneumothorax (red arrow).

**Fig. 4:**
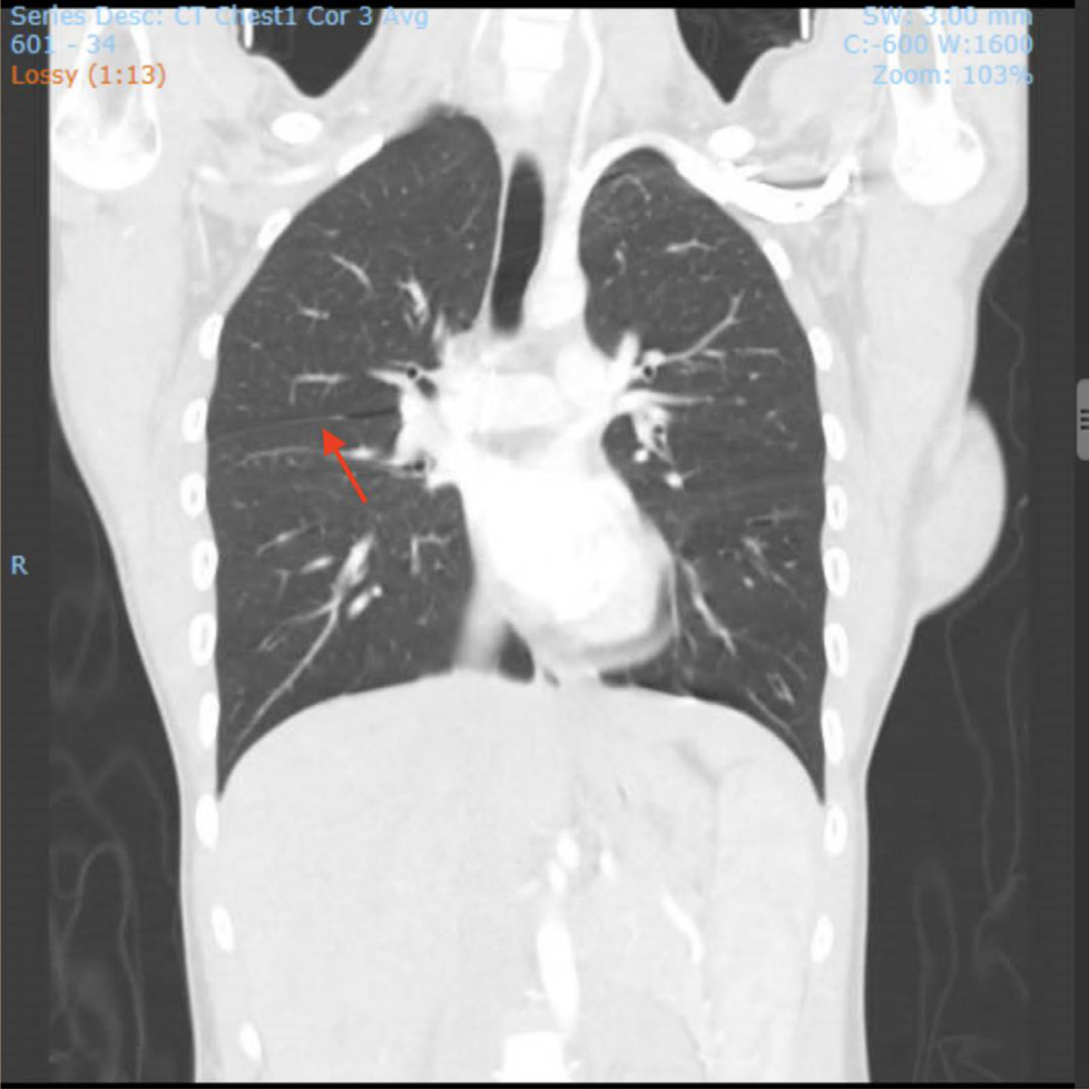
CT angiogram chest AP view showing the hyperlucency of primary spontaneous interlobar pneumothorax (red arrow).

Pulmonary embolism was one of the differential diagnoses and so a CT angiogram of the chest was performed in the ED to rule out pulmonary embolism, but no filling defects were seen (Fig. 2, Fig. 3, Fig. 4). However, a curvilinear streak was seen immediately adjacent to the course of the minor fissure of the right lung.

A diagnosis of primary spontaneous interlobar pneumothorax was made. The patient was treated with non-rebreather mask ventilation with 100% FiO2 overnight. Repeat chest X-ray did not show expansion of the pneumothorax. The patient remained clinically stable and was discharged home with a follow-up pulmonary clinic appointment. During her next appointment, a chest X-ray was done which did not show progression of pneumothorax nor did she have any clinical signs of pneumothorax.

## Discussion

With the advancement of medical diagnosis techniques, what would be initially diagnosed as a primary spontaneous pneumothorax may be later diagnosed as a secondary spontaneous pneumothorax after an underlying pulmonary disease is identified. However, spontaneous interlobar pneumothorax is a rare presentation of pneumothoraces.

Regardless of classification, presentation of a pneumothorax can range from asymptomatic to tension with hemodynamic instability. A patient presenting with mild symptoms of a pneumothorax can quickly progress to tension pneumothorax if not promptly identified and treated [3], [4], [5]. It most commonly presents with sudden shortness of breath and pleuritic chest pain. Signs depends on the size of pneumothorax, small ones are asymptomatic whereas large ones have decreased chest excursion on the affected side, enlarged hemithorax on the affected side, diminished breath sounds, absent tactile or vocal fremitus, and hyperresonant percussion [5]. Chest X-ray is often diagnostic for a larger pneumothorax, but if the amount of air in the pleural space is minimal, a dedicated CT scan may be required for identification.

The treatment of pneumothoraces depends on various factors, including the size of the pneumothorax and the presence of symptoms. Estimating the size of a pneumothorax remains a topic of debate, and there is currently no international consensus on the matter. The guidelines provided by the British Thoracic Society suggest measuring a pneumothorax from the chest wall to the lung edge at the level of the hilum [1]. According to these guidelines, a small pneumothorax is defined as being less than 2 cm, while a large pneumothorax is considered to be equal to or greater than 2 cm [1]. On the other hand, the American College of Chest Physicians recommends measuring a pneumothorax from the thoracic cupola to the lung apex [2]. According to their guidelines, a small pneumothorax is defined as being less than 3 cm, while a large pneumothorax is considered to be equal to or greater than 3 cm.

Clinically stable patients (<3 cm size of pneumothorax at apex according to American College of Chest Physicians) with a first episode of primary spontaneous pneumothorax that is small should be treated with observation with or without supplemental oxygen and discharged, if feasible [6]. Supplemental oxygen and observation is generally the strategy used in clinically stable patients with a first episode of primary spontaneous pneumothorax that is small and without severe symptoms [6]. A repeat chest X-ray 4-6 hours must be obtained. If the pneumothorax fails to improve or worsens, then the pleural air should be removed via catheter or chest tube thoracostomy [6].

## Conclusion

Spontaneous interlobar pneumothorax is a rare topographical finding and prompt diagnosis by a radiologist can help prevent complications and improve outcomes. While advancements in medical diagnosis techniques have improved the identification of underlying pulmonary disease, spontaneous interlobar pneumothorax remains a challenging diagnosis. Management of clinically stable patients with a first episode of primary spontaneous pneumothorax should involve observation, with intervention reserved for cases that fail to improve or worsen.

## Patient consent

Informed consent was obtained from the patient before writing up this case study
